# The Effects of Probiotics and Omega-3 Fatty Acids in Liver Steatosis Induced in Rats by High-Fructose Corn Syrup

**DOI:** 10.1155/2022/7172492

**Published:** 2022-01-31

**Authors:** Nildem Kizilaslan, Nihal Zekiye Erdem, Muzaffer Katar, Fikret Gevrek

**Affiliations:** ^1^Tokat Gaziosmanpasa University, Faculty of Health Sciences, Department of Nutrition and Dietetics, Tokat, Turkey; ^2^Istanbul Medipol University, School of Health Sciences, Department of Nutrition and Dietetics, Istanbul, Turkey; ^3^Tokat Gaziosmanpasa University, Faculty of Medicine, Department of Biochemistry, Tokat, Turkey; ^4^Tokat Gaziosmanpasa University, Faculty of Medicine, Department of Histology, Tokat, Turkey

## Abstract

**Aims:**

This study was designed to reveal the effect of probiotics and omega-3 fatty acids in a fatty liver model in rats induced by high-fructose corn syrup (HFCS).

**Methods:**

In the study, 40 male Wistar Albino rats were used, and these rats were divided into five groups. HFCS was added to the drinking water (30% solution) of four groups (Groups 2, 3, 4, and 5) for three weeks, and the animals were fed ad libitum. At the end of three weeks, the rats in Groups 3, 4, and 5 were administered omega-3 fatty acids (400 mg/kg) and probiotics (1.5 × 10^9^ cfu/mL/day) with the gavage method for four weeks. The body weights of rats were weighed and recorded before starting the experiment, at the end of the third week, and before the animals were sacrificed at the last week, all at the same hour. By subtracting the remaining amount of food and water from the daily food and water amount, the amount of food and water consumed was calculated. These values were recorded for seven weeks. At the end of the seven weeks, the rats were sacrificed after blood specimens and tissues were taken.

**Results:**

Analyzing the changes in the food intake of each group within itself throughout the experiment, it was observed that there was an increase in the food intake in the control group; from the starting week to the last week, the food intake amount of the HFCS group began to decrease particularly after the second week; and it began to decrease after the third week in the groups that were administered probiotics and omega-3 fatty acids. The changes in the sacrifice weights in the HFCS + omega-3 fatty acid, HFCS + probiotic, and HFCS + probiotic + omega-3 fatty acid groups were found to be lower than that in the HFCS group. The maximum levels of glucose, ALT, ALP, serum cholesterol, triglyceride and AST were found to be in the HFCS group. It was determined that the minimum mean steatosis level was in the control group, while the maximum steatosis level was in the HFCS group.

**Conclusions:**

As a result, there was a protective effect of probiotic and omega-3 fatty acid.

## 1. Introduction

Fatty liver, also known as hepatic steatosis, is a condition defined by excessive fat accumulation in the liver (>5% by weight) and by all clinical pictures where the diagnosis of steatosis is made due to any condition induced by alcohol or nonalcoholic factors [1]. The incidence and prevalence of nonalcoholic fatty liver disease (NAFLD) increase all around the world. According to the Global Guidelines of the World Gastroenterology Organization, the prevalence of NAFLD has doubled during the last 20 years. The figures indicate that this disease will be an increasingly common liver problem in both developed and underdeveloped countries, increasing the global burden of liver diseases, which will affect public health and lead healthcare costs to globally continue to increase. It is estimated that NAFLD/NASH will increase 5-year direct and indirect medical costs by 26% [2]. Studies have suggested that chronic disorders (obesity, diabetes, cardiovascular disease, hypertension, cancer, and metabolic syndrome) and fructose, in particular, may be associated with an increased prevalence of NAFLD. The “Fructose Hypothesis” has originated partially from animal studies and partially from historical trends. Particularly in animal experiments, it has been shown that high-fructose diets, compared to glucose, result in increased hepatic triglyceride content. Although the pathogenesis of the nonalcoholic fatty liver disease remains poorly understood, it is thought to be a multifactorial process involving genetic and environmental elements [3]. The first hit during the process of the disease which results in steatosis is insulin resistance. Cellular mechanisms, such as oxidative stress, mitochondrial dysfunction, and tumor necrosis factor-*α* (TNF*α*), and hormones, such as adiponectin and leptin, play a role in the second hit that involves inflammation and fibrosis [4]. It has been stated that excessive fructose may also play a role in the pathogenesis of NAFLD. Fructose is a highly lipogenic sugar and stimulates triglyceride synthesis. Perfusion studies show that fructose can stimulate the liver to produce higher levels of triglycerides in comparison to glucose [3].

At every stage of this disease, dietary habits should be emphasized rather than medical treatment. However, the effectiveness of lifestyle modification is low and decreases over time [5]. Therefore, there is a need to develop more effective and safe agents for this common disease. At present, there is no consensus for the treatment of NAFLD. Omega-3 fatty acids have recently been suggested as a potential treatment for NAFLD and show promise [6]. Probiotics may be used as effective biological factors for modulation of the intestinal microbiota, and studies demonstrated that they effectively improve liver functions. Recent studies reported that intestinal microbiota modulation has possible effects on the development of NAFLD/NASH [7, 8]. Intestinal flora colonized in healthy individuals may be influenced by many physiological and environmental factors such as diet, disease, aging, and stress. Administration of probiotics and omega-3 fatty acids as the recommended treatment for nonalcoholic fatty liver disease has initiated a new treatment method in this area. Thus, positive outcomes resulting from the presence of omega-3 fatty acids in the diet and probiotics to balance the intestinal flora have resulted in probiotics being recommended as a useful agent. However, the number of studies supporting this concept is very limited. This study aimed to reveal the effect of probiotics and omega-3 fatty acids in a fatty liver model in rats induced by high-fructose corn syrup.

## 2. Material and Method

Prior to the study, ethical approval (number: 17–9, date: 13.12.2018) was obtained from Gaziosmanpaşa University Animal Experiments Local Ethics Committee. The G*∗*Power 3.1.3 package program was used to calculate the sample size and conduct power analysis. This study was carried out at the Experimental Medicine Research Center (DETAB) of Tokat Gaziosmanpaşa University with 40 healthy, 8–12-week-old male Wistar Albino rats, weighing 200–250 g. In line with the approval obtained from Tokat Gaziosmanpaşa University Animal Experiments Local Ethics Committee, the principles of working with laboratory animals were adhered to throughout the study.

The rats were housed in a temperature-controlled (constant room temperature at 21 ± 2°C) environment with a 12 h light-dark cycle and ad libitum access to fresh tap water and standard laboratory chow diet. The rats were randomly divided into five groups (8 rats per group).

### 2.1. Groups and Food Intake

The rats were divided into five groups and monitored for seven weeks.

  Group 1 (control group): these rats were fed only standard laboratory chow and water during the study.
  Group 2 (high-fructose corn syrup (HFCS) group): high-fructose corn syrup (55% fructose, 45% glucose) was provided and added to the drinking water. These rats were fed standard laboratory chow and water during the study.
  Group 3 (high-fructose corn syrup (HFCS) + omega-3 fatty acid): high-fructose corn syrup (55% fructose, 45% glucose) was provided in the drinking water, and 400 mg/kg omega-3 fatty, acid omega-3 950 (Solgar Inc.), was provided with the gavage method. These rats were fed standard laboratory chow and water during the study.
  Group 4 (high-fructose corn syrup (HFCS) + probiotic): high-fructose corn syrup (55% fructose, 45% glucose) was provided in the drinking water, and 1.5 × 10^9^ cfu/mL/day (VSL#3 probiotic preparation (Alfasigma USA, Inc.) was provided with the probiotic gavage method, to contain live probiotic bacteria. These rats were fed standard laboratory chow and water during the study.
  Group 5 (high-fructose corn syrup (HFCS) + probiotic + omega-3 fatty acid): high-fructose corn syrup (55% fructose, 45% glucose) was provided in the drinking water, and 400 mg/kg omega-3 fatty acid and 1.5 × 10^9^ cfu/mL/day probiotics were provided with the probiotic gavage method, to contain live probiotic bacteria. These rats were fed standard laboratory chow and water during the study.

Throughout the study, all groups were fed with standard laboratory chow and water. By subtracting the remaining amount of chow and water from the daily chow and water amount, the amount of chow and water consumed was calculated, and these values were recorded for seven weeks. The body weights of the rats were calculated and recorded before starting the experiment, at the end of the third week, and before the animals were sacrificed in the last week, all at the same hour. High-fructose corn syrup was added to the drinking water (30% solution) of four groups (Groups 2, 3, 4, and 5) for three weeks, and the animals were fed ad libitum. HFCS was used to create the model. Considering the studies in the literature, it was thought that it would be appropriate to use HFCS to create a model [9]. At the end of three weeks, the rats in Groups 3, 4, and 5 were administered omega-3 fatty acids and probiotics with the gavage method for four weeks. The used dose is obtained from the references. To produce the same stress for Groups 1 and 2, 0.9% sodium chloride (NaCl) was provided with the 0.2 cc gavage method. The administrations of probiotics, omega-3 fatty acids, and normal saline were performed at the same hour every day and following the same group order. At the end of the seven weeks, the rats were sacrificed after blood specimens and tissues were taken.

### 2.2. Data Collection and Measurement Methods

At the end of the seven-week study, for sacrificing them, the rats' weights were recorded, and the rats were anesthetized with an intraperitoneal injection of a combination of 100 mg/kg ketamine and 10 mg/kg xylazine. Anesthetic depth was monitored by applying stimuli to the rats' tails. Blood specimens were taken from the rats using 5 cc syringes with the intracardiac method, the abdominal region of the rats was sterilized with an antiseptic solution (BATTICON solution), and the surgical procedure proceeded. The liver was immediately removed and weighed on a microbalance. Half of the liver was placed in a 10% formalin solution for histology. The other half was wrapped in aluminum foil for biochemical parameters and stored at −80°C.

### 2.3. Examination of Biochemical Parameters

At the end of the seventh week, the animals were anesthetized with a combination of ketamine hydrochloride (100 mg/kg) and xylazine hydrochloride (10 mg/kg), blood specimens were taken with the intracardiac method, and the animals were sacrificed. Blood specimens were collected in EDTA tubes and gel-coated dry tubes and then centrifuged at 4400 rpm for 10 minutes at 4 °C. The serums obtained from the blood specimens were sent to the laboratory on the same day.

In the laboratory, serum glucose, total cholesterol (TC), triglyceride (TG), alanine aminotransferase (ALT) and aspartate aminotransferase (AST) (liver enzymes), and alkaline phosphatase (ALP) levels were measured in the serum samples belonging to all groups. These biochemical parameters were studied in a Roche Cobas C501 analyzer and with Roche kits.

### 2.4. Histopathological Examination of Samples

Liver tissues were kept in 10% formalin solution for 1 day. After fixation, the trimming process was applied. After 24 hours, the washing procedure was started. Tissues were placed in 70%, 80%, 96%, and 100% alcohol, respectively, for 1 h. Tissues were routinely followed and placed in paraffin-embedded tissue sections.  5*μ*m sections were taken from the paraffin blocks and stained with hematoxylin and eosin. The findings were examined under a light microscope (Olympus CX21-Japan) and photographs were taken. Histopathologic scoring was made according to the scoring system developed by Kleiner's group. Histopathological examinations were blinded.

### 2.5. Protocol for Hematoxylin-Eosin in Liver Tissue

Deparaffinization: tissues were deparaffinized in 3 different xylenes for 10 min each. Dehydration: tissues were kept in 100%, 90%, 80%, and 70% alcohol, respectively, for 5 min. Washing: tissues were washed with distilled water for 5–10 min. Mayer's hematoxylin phase: tissues were kept for 10 min. Washing: tissues were rewashed with distilled water for 5–10 min. Eosin phase: tissues were kept for 3 min. Washing: tissues were rewashed with distilled water for 5–10 min. Alcohol phase: tissues were kept in 70% and 80% alcohol for 1 min and in 90% and 100% alcohol for 2 min, respectively. Xylene phase: tissues were kept in 3 different xylenes for 5 min. Adhesion: tissues in xylene were taken for the adhesion process. They were removed one by one, adhered with synthetic resin (Entellan), and left to dry.

### 2.6. Statistical Methods Applied in Data Analysis

The data are expressed as descriptive values, arithmetic mean ± standard deviation, and minimum and maximum values. In data analysis, the Kolmogorov–Smirnov test was performed to determine whether the series were normally distributed. According to this test, an independent-samples *t*-test was applied in the analysis of two independent groups with normal distribution, while a Mann–Whitney test was applied in the analysis of the nonnormally distributed series. While comparing three or more groups, a one-way ANOVA test was used in the normally distributed series, whereas Kruskal–Wallis H-test was used in the nonnormally distributed series. In the comparison of the pre- and postmeasurements made/taken of a group, paired-samples *t*-test was used for the normally distributed series, while a Wilcoxon test was used for the nonnormally distributed series. Correlation analysis was performed to determine whether or not there was a linear relationship between two numerical measurements, and the direction and severity of this relationship if any. The obtained analysis results were evaluated and interpreted on the significance levels of *p* ≤ 0.05, *p* ≤ 0.01, and *p* ≤ 0.001.

## 3. Results

### 3.1. Analysis of Changes regarding Food Intake Levels of Rats

**Figure 1:**
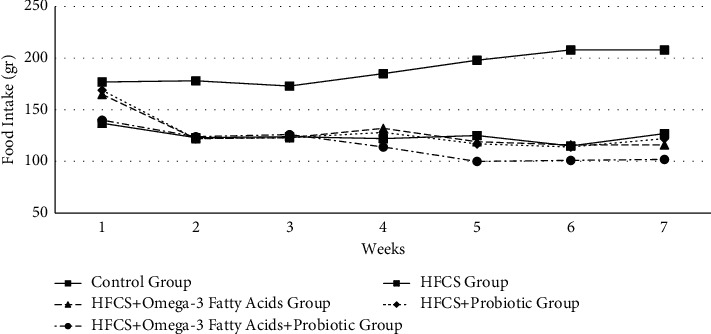
Changes in weekly food intake amount.

The changes in the amount of food intake of the rats by the groups are given in Tables 1 and 2and Figure 1. As shown in Tables 1 and 2, the differences in the weekly food intake of the groups during the experiment were found to be statistically significant (*p* ≤ 0.001). Analyzing the changes in the food intake of each group within itself during the experiment, an increase in the food intake in the control group was observed from the starting week to the last week. The increase in the amount of food intake seen in the control group in the periods of weeks 3-4, weeks 4-5, and weeks 5-6 was found significant (*p* ≤ 0.05). The amount of food intake in the HFCS group began to decrease especially after the second week, while the reduction in the amount of food intake between weeks 1 and 2 was found to be significant (*p* ≤ 0.05). The amount of food intake in the HFCS + omega-3 fatty acid group, the HFCS + probiotic group, and the HFCS + probiotic + omega-3 fatty acid group decreased even more depending on the omega-3 and probiotic administrations made to the rats after the third week. The decrease in the amount of food intake of the HFCS + omega-3 fatty acid group in weeks 4 and 5 was found significant (*p* ≤ 0.05). Again, the decrease in the amount of food intake in both the HFCS + probiotic and HFCS + probiotic + omega-3 fatty acid groups between weeks 3 and 4 and between weeks 4 and 5 was found significant (*p* ≤ 0.05).

### 3.2. Analysis of Changes regarding Fluid Intake Levels of Rats

**Figure 2:**
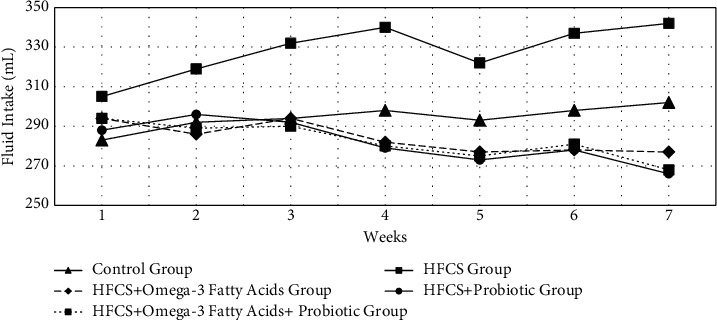
Changes in weekly fluid intake amounts.

The changes in the amount of weekly fluid intake of the rats by the groups are given in Table 3 and Figure 2. As seen in Table 3, the difference in the first-week fluid intake amounts of the rats by the groups was statistically insignificant (*p* ≥ 0.05). In the first week, the fluid intake of the HFCS group was found to be significantly higher in comparison to the control group (*p* ≤ 0.01). Moreover, after the first week, the fluid intake amounts during the experiment between weeks 2 and 7 by the groups were significantly different (*p* ≤ 0.01). The analysis revealed that there was an increase in the fluid intake amounts of the control group by the weeks, whereas this increase was statistically insignificant (*p* ≥ 0.05). The changes in the fluid intake amounts of the HFCS group in the periods of weeks 1-2, 2-3, 3-4, and 5-6 were found significant (*p* ≤ 0.05). Throughout the experiment, the fluid intake amounts decreased by weeks in the groups of HFCS + omega-3 fatty acid, HFCS + probiotic, and HFCS + probiotic + omega-3 fatty acid. However, the reduction in these groups between weeks 1 and 7 was statistically insignificant (*p* ≥ 0.05).

### 3.3. Analysis of Changes in Body and Liver Weights of Rats

**Figure 3:**
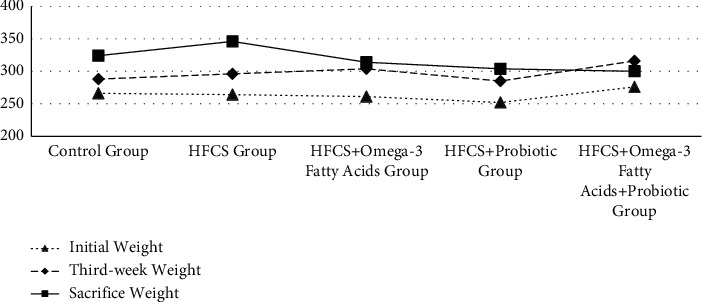
Initial, third week, and sacrifice weights of rats (gr).

**Figure 4:**
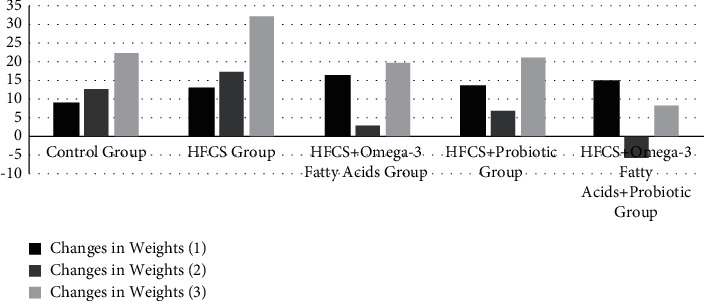
Changes in weights of rats (%).

**Figure 5:**
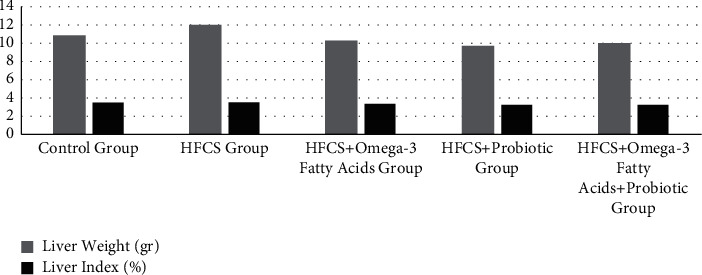
Liver weights and liver index values of rats.

The body and liver weights and liver indices of the rats are given in Table 4 and Figures 3–5. The initial, third week, and sacrifice weights of the rats were not significantly different based on the groups (*p* ≥ 0.05). To observe the change in the body weights of the rats during the experiment, weight change was calculated using three different methods. As a result of the analysis, the first, second, and third measurement weight changes were significantly different among the groups (*p* ≤ 0.001). In terms of the first weight change, it was observed that the minimum change was in the control group (8.67%), while the maximum change was in the HFCS + omega-3 fatty acid group (16.38%). In terms of the second weight change, it was observed that the minimum change was in the HFCS + probiotic + omega-3 fatty acid group (-5.67%), while the maximum change was in the HFCS group (17.15%). In terms of the third weight change, it was observed that the minimum change was in the HFCS + probiotic + omega-3 fatty acid group (8.15%), while the maximum change was in the HFCS group (31.96%). In terms of the liver weights of the rats, the minimum values were found in the HFCS + probiotic group, while the maximum values were found in the HFCS group. The difference among all groups in terms of liver weight was significant (*p* ≤ 0.05). The liver weights were found to be significantly lower in the groups of HFCS + omega-3 fatty acid, HFCS + probiotic, and HFCS + probiotic + omega-3 fatty acid in comparison to the HFCS group (*p* ≤ 0.01). In the analysis performed, no statistically significant difference was found among the groups in terms of the liver index (*p* ≥ 0.05). It was determined that the HFCS + probiotic group had the minimum liver index, while the HFCS group had the maximum liver index.

### 3.4. Glucose, Liver Enzymes, Serum Cholesterol, and Triglyceride Contents of Rats by Groups

**Figure 6:**
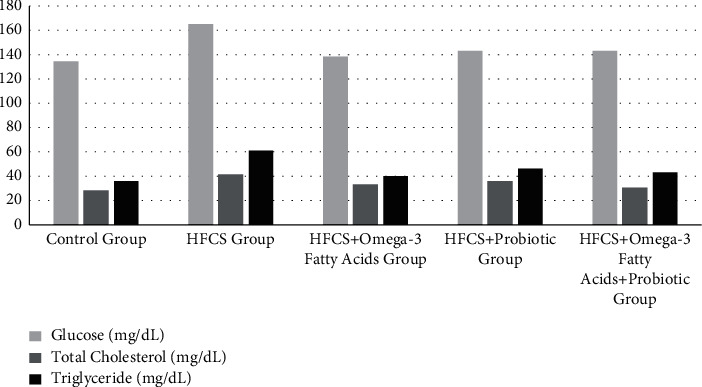
Glucose, serum cholesterol, and triglyceride levels of rats.

**Figure 7:**
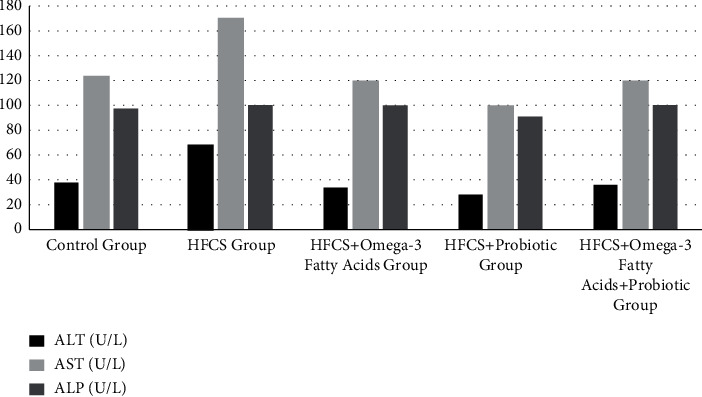
Liver enzyme levels of the rats.

The glucose, liver enzymes, serum cholesterol, and triglyceride levels of the rats are given in Table 5 and Figures 6 and 7. Comparing the mean glucose levels of the groups, the difference among the groups was found to be statistically significant (*p* ≤ 0.05). It was found that the minimum glucose level was in the control group (132.98 ± 21.09 mg/dL), while the maximum glucose level was in the HFCS group (164.73 + 15.78 mg/dL). In the analysis performed to determine from which group the difference between the groups originated, the mean glucose level of the HFCS was significantly higher than the control group (*p* ≤ 0.01). Analyzing the difference between the groups, the mean ALT level of the HFCS + probiotic group was significantly lower than the control group (*p* ≤ 0.05). The mean ALT levels were significantly lower in the HFCS + omega-3 fatty acid, HFCS + probiotic, and HFCS + probiotic + omega-3 fatty acid groups compared to the HFCS group (*p* ≤ 0.05). The ALT level of the HFCS + probiotic + omega-3 fatty acid group was significantly higher than the HFCS + probiotic group (*p* ≤ 0.05). Comparing the mean AST levels of the groups, the difference among the groups was statistically significant (*p* ≤ 0.01). The mean AST level was significantly higher in the HFCS group than in the control group (*p* ≤ 0.05). The mean AST levels of the HFCS + omega-3 fatty acid, HFCS + probiotic, and the HFCS + probiotic + omega-3 fatty acid groups were significantly lower than the HFCS group (*p* ≤ 0.001). The difference among the mean ALP values of the groups was statistically insignificant (*p* ≥ 0.05). It was found that the minimum ALP value was in the HFCS + probiotic group (90.25 + 16.85 U/L), while the maximum ALP value was in the HFCS group (99.12 + 23.28 U/L). The difference among the mean serum total cholesterol levels of the groups was statistically significant (*p* ≤ 0.001). In the analysis performed to determine from which group the difference between the groups originated, the serum total cholesterol levels of the HFCS group and the HFCS + probiotic group were found to be significantly higher than the control group (*p* ≤ 0.05). The total cholesterol levels of the HFCS + omega-3 fatty acid, HFCS + probiotic, and HFCS + probiotic + omega-3 fatty acid groups were significantly lower than the HFCS group (*p* ≤ 0.01). The difference among the mean serum total triglyceride levels of the groups was statistically significant (*p* ≤ 0.05). In the analysis performed, the serum triglyceride level of the HFCS group was significantly higher than the control group (*p* ≤ 0.01). The mean serum total triglyceride levels of the HFCS + omega-3 fatty acid, HFCS + probiotic, and HFCS + probiotic + omega-3 fatty acid groups were significantly lower than the HFCS group (*p* ≤ 0.05).

### 3.5. Liver Tissue Triglycerides, Total Cholesterol, and Total Protein Contents of Rats by Groups

Liver tissue triglycerides, total cholesterol, and total protein contents by experimental groups are given in Table 6. The minimum mean tissue triglyceride level was in the control group (6.08 ± 0.91 *μ*mol/g tissue), while the maximum mean tissue triglyceride level was in the HFCS group (11.16 ± 1.55 *μ*mol/g tissue). The difference between the groups in terms of liver tissue mean triglyceride levels was found to be statistically significant (*p* ≤ 0.001). It was determined that the minimum mean tissue cholesterol level was in the control group (1.78 ± 0.63 *μ*mol/g tissue), while the maximum mean tissue cholesterol level was in the HFCS group (3.23 ± 0.39 *μ*mol/g tissue). The difference between the liver tissue total cholesterol levels of the experimental groups was found to be statistically significant (*p* ≤ 0.001). It was observed that the minimum mean tissue protein level was in the HFCS + probiotic group (5.61 ± 0.80 mg/g protein), while the maximum mean tissue protein level was in the HFCS group (8.02 ± 1.05 mg/g protein). The difference between the liver tissue total protein levels of the experimental groups was found to be statistically significant (*p* ≤ 0.01).

### 3.6. Histopathologic Findings

**Figure 8:**
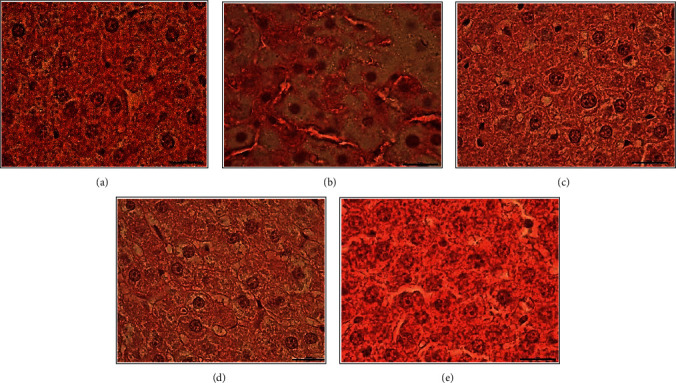
Control group (a), HFCS group (b), HFCS + omega-3 fatty acids group (c), HFCS + probiotic group (d), and HFCS + omega-3 fatty acids + probiotic group (e) (*H* + *E*; 100x).

**Figure 9:**
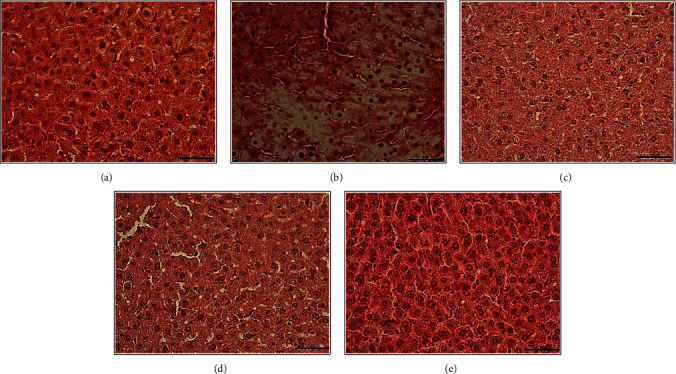
Control group (a), HFCS group (b), HFCS + omega-3 fatty acids group (c), HFCS + probiotic group (d), and HFCS + omega-3 fatty acids + probiotic group (e) (*H* + *E*; 40x).

Microscopic scoring values for the hepatic steatosis in liver tissue by experimental groups are given in Table 7. It was determined that the minimum mean steatosis level was in the control group (0.18 ± 0.11), while the maximum steatosis level was in the HFCS group (2.05 ± 0.17) (Figures 8 and 9). The difference between the groups in terms of steatosis levels was found to be statistically significant (*p* ≤ 0.001). While no lobular inflammation was observed in the control group, the maximum lobular inflammation level was in the HFCS group (1.08 ± 0.12) (Figures 8 and 9). The difference between the groups in terms of lobular inflammation levels was statistically significant (*p* ≤ 0.001). Hepatocellular ballooning was not monitored in the control, HFCS + omega-3 fatty acid, HFCS + probiotic, and HFCS + probiotic + omega-3 fatty acid groups (Figures 8 and 9). Hepatocellular ballooning level of the HFCS group was found as 0.27 ± 0.06. Hepatocellular ballooning levels were found to be lower in the control, HFCS + omega-3 fatty acid, HFCS + probiotic, and HFCS + probiotic + omega-3 fatty acid groups, compared to the HFCS group, while the difference was statistically significant (*p* ≤ 0.001). The minimum mean NAS level was in the control group (0.18 ± 0.11), while the maximum NAS level was in the HFCS group (3.41 ± 0.24). The difference between the groups in terms of NAS levels was statistically significant (*p* ≤ 0.001). Given the histologic picture based on these results, it was observed that the livers of the other groups except for the HFCS group were histologically normal (Figures 8 and 9).

## 4. Discussion

It was observed that the use of probiotics in humans increases the effectiveness of lifestyle modifications in obese individuals with NAFLD, can improve conventional liver function tests, and may reduce markers for lipid peroxidation. However, sufficient data could not be provided on this topic. Although there are encouraging studies on omega-3 fatty acids, the number of studies is very limited. Additionally, even though there are limited studies in the current literature separately examining the effects of probiotics and omega-3 fatty acid supplements in NAFLD, there has been no study that investigated their effects together. The main purpose of this approach was to observe the effect of two supplements, both histologically and anthropometrically. At this point, the discussion on the effects of high-fructose corn syrup, omega-3 fatty acids, and probiotics on fatty liver in terms of food intake and biochemical parameters has started.

The content of fast food or cafeteria-style foods is based on saturated fats, cholesterol, and fructose [9]. It is known that excessive consumption of fructose may lead to an increase in de novo lipogenesis [10]. In particular, since it has been added to the composition of almost all sweet foods in recent years, the effect of fructose on health began to be questioned, and the number of studies on this topic has increased. In studies, it has been aimed to determine the relationship between consumption of high-fructose corn syrup and excessive fructose and factors adversely affecting human health such as obesity, coronary diseases, adverse metabolic changes, increased plasma triglyceride levels, and hepatic insulin resistance [11]. Fructose is reported to lead to insulin resistance, dyslipidemia, proinflammatory cytokines, and hepatic lipid peroxidation. Therefore, rats fed fructose-enriched diets are widely recognized as good models for NAFLD and NASH [12]. Studies have shown that consumption of high-fructose corn syrup is common in these patients. Hence, high-fructose corn syrup was used in this study for the fatty liver model [13, 14].

Chen et al. stated that the energy intake and body weights of rats treated with high-fructose corn syrup significantly increased compared to the control group [15]. In their study carried out on rats fed with a high-fructose diet for nine weeks, Masterjohn et al. reported that the body weights of the rats in the high-fructose diet group increased at the end of the study [16]. In their randomized study, Alisi et al. revealed a significant reduction in the BMI of children with NAFLD, whose fatty liver severity was examined with ultrasound and who were treated with *Bifidobacterium*, lactobacilli 5, and *S. thermophilus* strains (VSL # 3) for 4 months. These data showed that these strains can reduce fatty liver and thus prevent the progression of NAFLD [17]. In the animal models of Cani et al., treatment with oligofructose led to a decrease in the glucose tolerance and body weight of patients with NAFLD [18].

In this study, the changes in the sacrifice weights in the HFCS + omega-3 fatty acid, HFCS + probiotic, and HFCS + probiotic + omega-3 fatty acid groups were lower than that in the HFCS group. The weight change in the HFCS group in the process from the initial weight to the sacrifice weight was found as 31.96%. The other groups remained on the level of the control group (22.45%). Only the weight of the HFCS + probiotic + omega-3 fatty acid group increased by 8.15%. This result suggested that HFCS may have adverse effects on human health, and supplements may affect this process in a protective manner. It is thought that HFCS may cause obesity, which may increase the hepatic steatosis level in the future.

In a study investigating the relationship between fructose and appetite, Bellisle et al. determined that fructose intake affects the mechanism of appetite [19]. Chen et al. found that the food and fluid intake of rats treated with high-fructose corn syrup was significantly lower than the control group [15]. Panchal et al. reported that daily average food and fluid intake of rats administered high levels of fructose decreased throughout 16 weeks compared to the control group [20]. Narayanan et al. stated no difference between the food and fluid intake amounts of rats fed with high-fructose corn syrup and the control group [21]. Masterjohn et al. concluded that there was no difference between the food intake amounts of rats fed with a high-fructose diet for nine weeks and the control group [16]. In their study, Unsal et al. observed that rats fed high-fructose corn syrup consumed more fluids, but the same groups consumed less feed. They concluded that they met their calorie needs from HFCS, so they moved away from healthy eating [22]. In this study, the differences in the weekly food intake amounts of the groups during the experiment were statistically significant. Analyzing the changes in food intake of each group throughout the experiment, it was observed that there was an increase in the food intake amount of the control group from the initial week to the last week. The food intake amount of the HFCS group began to decrease especially after the second week. In contrast, the reduction in the food intake amount between weeks 1 and 2 was statistically significant. The food intake amounts in the HFCS + omega-3 fatty acid, HFCS + probiotic, and HFCS + probiotic + omega-3 fatty acid groups decreased even more after the third week, depending on the administration of omega-3 and probiotics to the rats, and this change was statistically significant. Consumption of drinking water with high-fructose content resulted in the reduction of food intake, whereas it was thought to cause an increase in energy intake. This situation showed parallel results with the change in body weight. This supported the hypothesis of the study.

In this study, the change in the fluid intake amounts in the HFCS group was statistically significant between weeks 1 and 2, 2 and 3, 3 and 4, and 5 and 6. Throughout the experiment, the fluid intake amount decreased by weeks in the groups of HFCS + omega-3 fatty acid, HFCS + probiotic, and HFCS + probiotic + omega-3 fatty acid. However, the reduction in these groups between weeks 1 and 7 was statistically insignificant. This result was thought to indicate the reason for the reduction in the food intake and the change in the amount of the fluid intake of the HFCS group in comparison to the other groups. It was concluded that the tendency towards high-fructose drinking water may have increased hepatic steatosis content, along with unhealthy diets. The change in fluid intake to decrease especially after the gavage method in the other groups may have been due to the finding that these supplements showed positive results.

In their study on omega-3 fatty acids, Zhu et al. included 144 patients with hyperlipidemia-induced NAFLD in a randomized controlled study for 24 weeks. The patients were randomly divided into two groups. Group A (*n* = 72) took the prescribed diet and 2 g omega-3 PUFA three times a day. Group B (*n* = 72) took the prescribed diet and 2 g of placebo three times a day. As a result, it was concluded that omega-3 PUFAs were safe and effective for patients with hyperlipidemia-induced NAFLD, and they reduced ALT and serum lipid levels to normal levels [23]. In their study conducted in Italy, Loguercio et al. examined the effects of the chronic use of probiotics in 22 NASH, 20 ASH, and 36 HCV patients (20 CHC, 16 cirrhosis). All patients were administered the VSL#3 probiotic. AST and ALT levels were significantly normalized after the VSL#3 probiotic treatment in the NASH, ASH, and chronic hepatitis C cases [24]. Aller et al. measured the effects of *L. bulgaricus* and *S. thermophilus* on different parameters to investigate liver functions and cardiovascular risk factors. This treatment showed a decrease in ALT, aspartate aminotransferase (ASP), and *γ*-GTP levels [25]. In the study of Wong et al., NASH patients used Lepicol probiotics for 6 months. The probiotic treatment reduced fatty liver and AST levels [26].

Nogueira et al. divided 60 NAFLD patients into two groups. 27 patients took 0.315 g/day omega-3 PUFA. 23 patients were grouped as the placebo group. It was concluded that supplementation with omega-3 PUFA significantly affected the NAFLD patients' lipid profiles, potential levels of omega-6 fatty acids, and serum triglyceride levels [27]. In their double-blind, randomized controlled clinical trial, Nabavi et al. provided NAFLD patients with 300 gr *L. acidophilus* La5 and *B. lactis* Bb12 and probiotic yogurt daily for eight weeks. The consumption of *L. acidophilus* La5 and *B. lactis* Bb12 provided a reduction in ALT, ASP, TC, and LDL-C serum levels in comparison to the control group [28].

In this study, analyzing the mean serum levels of the experimental groups, the difference among the groups was statistically significant. The maximum ALT, serum cholesterol, and AST levels were in the HFCS group. The mean ALT, AST, and serum cholesterol values were significantly lower in the groups of HFCS + omega-3 fatty acid, HFCS + probiotic, and HFCS + probiotic + omega-3 fatty acid groups in comparison to the HFCS group. Abnormal AST and ALT levels in the clinic may signal liver damage. This may be a histopathological indicator of NAFLD. This situation in the other groups was based on the hypothesis that probiotic and omega-3 fatty acids exhibit anti-inflammatory and antioxidant activity.

In the study of Qin et al., 80 patients with NAFLD associated with hyperlipidemia were randomly assigned to consume fish oil (*n* = 40, 4 g/d) or corn oil capsules (*n* = 40, 4 g/d). Fish oil supplements (4 g) for 3 months improved lipid and glucose levels, liver function, and the circulating biomarkers and performed anti-inflammation functions in patients with NAFLD associated with hyperlipidemia. They suggested that supplementation with fish oil can have benefits in the treatment of metabolic abnormalities associated with NAFLD [29]. In this study, too, it was found that the minimum mean tissue triglyceride level was in the control group, while the maximum mean tissue triglyceride level was in the HFCS group. Tissue triglyceride level in the HFCS + omega-3 fatty acid, HFCS + probiotic, and HFCS + probiotic + omega-3 fatty acid groups were lower than the HFCS group, while the difference was statistically significant. This result supports that the HFCS increases hepatic steatosis content. Lower levels of triglycerides in other groups despite the continuation of a high-fructose diet suggests that probiotic and omega-3 fatty acids improve the lipid profile.

Lee et al. reported that a diet based on high cholesterol, high saturated fat, and high-fructose recapitulates all features of the NAFLD, including metabolic syndrome and NASH with progressive fibrosis [30]. In their study, Tetri et al. added 55% fructose and 45% glucose to drinking water (42 g/l) of mice. Insulin resistance, obesity, and severe hepatic steatosis have developed [31]. Ma et al. divided C57BL-6 mice into two groups. The first group was administered a high-fat diet (60%), while the second group was given standard pellet feed. Mice received VSL#3 probiotic (1,5 × 10^9^ CFU/mouse/day) with gavage method during 4 weeks. Probiotic treatment provided significant improvement in steatosis and insulin resistance [32]. Velayudham et al. divided 16 C57BL-6 mice into two groups. Both groups were fed with a methionine-choline-deficient (MCD) diet during 6 weeks. One group was also administered VSL#3 probiotic. One packet of VSL#3 (450 billion colonies/packet) was mixed in 1L of water and provided to mice. VSL#3 probiotic ameliorated liver fibrosis [33]. In the study of Nogueira et al., 27 patients were administered 0.315 gr/day omega-3 PUFA. Plasma increase of n-3 PUFAs (especially DHA) was determined to be associated with better liver histology [34]. There is evidence that omega-3 suppresses and decreases hepatic TG accumulation, and thus, it reduces the NAFLD. However, the clinical evidence for the benefits of omega-3 in NAFLD have not been stated [35]. Experimental studies have shown that diets enriched with omega-3 fatty acids improve insulin sensitivity [36] and reduce intrahepatic triglyceride content and steatohepatitis in rats [37]. Konuma et al. investigated the effect of EPA on MC4R-KO mice. They demonstrated that EPA treatment effectively prevents the development and progression of liver fibrosis in MC4R-KO mice, and hepatic steatosis reduced significantly. This study unravels a novel antifibrotic mechanism of EPA, thereby suggesting a clinical implication for the treatment of NASH [38]. While no lobular inflammation was observed in the control group, it was determined that the HFCS group had the maximum level of lobular inflammation. The difference between the groups in terms of lobular inflammation levels was statistically significant. No hepatocellular ballooning was observed in the control, HFCS + omega-3 fatty acid, HFCS + probiotic, and HFCS + probiotic + omega-3 fatty acid groups. This result may highlight inconsistent results on omega-3 PUFAs and probiotics in terms of steatosis. It was concluded that omega-3 fatty acids and probiotics suppress and reduce triglyceride accumulation. These supplements are thought to have protective properties in terms of steatosis. It is thought that probiotics and omega-3 supplements given single are also effective, but combined support is more effective in protecting against steatosis.

## 5. Conclusion

From a public health perspective, due to the rapid growth of fatty liver disease worldwide and the increasing use of high-fructose corn syrup, especially in the composition of foods, this disease becomes alarming every passing day. Today, the fundamental grounds of treatment are based on nutrition therapy, weight loss, and exercise. However, these interventions fail to be permanent, and their effectiveness is low. Omega-3 fatty acids and probiotics have recently taken their place among the treatment methods discussed for this disease. Although there are limited studies in the current literature separately examining the effect of probiotics and omega-3 fatty acid supplements in NAFLD, no study investigated their effects together. In the high-fructose diet model, these supplements were found to have a useful effect on some serum parameters and appetite control that are important in fatty liver disease. The interaction between the gut-liver axis, dietary factors, microbiota and intestinal barrier integrity, and a high-fructose diet and an omega-3 fatty acids-deficient diet are thought to play an important role in the development of NAFLD. Omega-3 fatty acids and probiotics reduce and even stop the progression of steatosis. It was observed in a high-fructose diet model that these supplements have a beneficial effect on some liver tissue parameters important in fatty liver and on weight management. This study also highlights the importance of reversing the histopathologic modifications in the liver. It was demonstrated that administration of a supplement with probiotics and omega-3 fatty acids reduced hepatic steatosis content, and it appears to be a useful treatment in NAFLD, especially when combined with nutritional intervention. Omega-3 fatty acids and probiotics are safe, well-tolerated, and usually do not have negative side effects. This study indicated that, in a fatty liver model induced by high-fructose corn syrup, omega-3 fatty acids and probiotics can provide protection against the side effects of high-fructose levels on the liver. It is therefore thought that omega-3 fatty acid and probiotic supplements in NAFLD may be an alternative approach for the remission of the disease. To evaluate the benefits of omega-3 fatty acids and probiotics completely, there is a need for a longer period, sufficient-sized, and appropriately controlled randomized clinical trials.

## Figures and Tables

**Table 1:** Nonalcoholic fatty liver disease scoring system [10].

Steatosis		Lobular Inflammation		Hepatocellular ballooning	
0	<%5	0	0 no foci	0	None
1	%5–33	1 field	<2 foci per ×200 field	1	Few balloon cells
2	%34–66	2 fields	2–4 foci per ×200 field	2	Many cells/prominent ballooning
3	>%66	3 fields	>4 foci per ×200 field		

**Table 2:** Weekly food intake levels in rats.

Food intake (g)						
Weeks	Control group (*n* = 8)	HFCS group (*n* = 8)	HFCS + omega-3 fatty acids group (*n* = 8)	HFCS + probiotic group (*n* = 8)	HFCS + omega-3 fatty acids + probiotic group (*n* = 8)	*p* ^*∗*^
	Mean ± SD	Mean ± SD	Mean ± SD	Mean ± SD	Mean ± SD	
1	177.71 ± 16.15	137.14 ± 12.88^a3^ ^*∗*^ ^*∗*^	165.14 ± 11.12^b3^ ^*∗*^ ^*∗*^	168.57 ± 13.85^b3^ ^*∗*^ ^*∗*^	140.00 ± 13.74^a3.c3.d3^ ^*∗*^ ^*∗*^	**0.001**
2	178.00 ± 15.88	123.00 ± 11.74^a3^ ^*∗*^ ^*∗*^	122.00 ± 11.86^a3^ ^*∗*^ ^*∗*^	122.42 ± 12.73^a3^ ^*∗*^ ^*∗*^	124.00 ± 12.16^a3^ ^*∗*^ ^*∗*^	**0.002**
*p* ^*∗∗∗*^	0.680	**0.034**	**0.018**	**0.018**	**0.028**	
3	173.28 ± 14.20	123.71 ± 11.02^a3^ ^*∗*^ ^*∗*^	122.57 ± 12.09^a3^ ^*∗*^ ^*∗*^	122.53 ± 12.83^a3^ ^*∗*^ ^*∗*^	125.71 ± 13.68^a3^ ^*∗*^ ^*∗*^	**0.002**
*p* ^*∗∗∗*^	0.114	0.785	0.705	0.931	0.083	
4	185.00 ± 14.98	122.00 ± 10.34^a3^ ^*∗*^ ^*∗*^	132.00 ± 11.97^a3.b1^ ^*∗*^ ^*∗*^	128.28 ± 11.95^a3^ ^*∗*^ ^*∗*^	113.85 ± 12.15^a3.c1.d1^ ^*∗*^ ^*∗*^	**0.001**
*p* ^*∗∗∗*^	**0.018**	0.680	0.108	**0.043**	**0.049**	
5	198.57 ± 14.04	125.14 ± 11.78^a3^ ^*∗*^ ^*∗*^	118.57 ± 13.77^a3^ ^*∗*^ ^*∗*^	117.28 ± 12.24^a3^ ^*∗*^ ^*∗*^	100.14 ± 12.66^a3.b3.c1.d1^ ^*∗*^ ^*∗*^	**0.001**
*p* ^*∗∗∗*^	**0.016**	0.109	**0.017**	**0.028**	**0.043**	
6	208.85 ± 12.66	114.85 ± 11.85^a3^ ^*∗*^ ^*∗*^	116.28 ± 13.63^a3^ ^*∗*^ ^*∗*^	113.57 ± 11.07^a3^ ^*∗*^ ^*∗*^	101.00 ± 12.05^a3.c1^ ^*∗*^ ^*∗*^	**0.001**
*p* ^*∗∗∗*^	**0.014**	0.063	0.336	0.733	0.414	
7	208.14 ± 12.53	126.85 ± 12.83^a3^ ^*∗*^ ^*∗*^	115.71 ± 13.94^a3^ ^*∗*^ ^*∗*^	117.85 ± 7.78^a3^ ^*∗*^ ^*∗*^	101.71 ± 10.73^a3.b3.c1.d3^ ^*∗*^ ^*∗*^	**0.001**
*p* ^*∗∗∗*^	0.680	0.091	0.593	0.464	0.799	

^*∗*^Kruskal–Wallis H-test.  ^*∗*^ ^*∗*^Mann–Whitney *U* test.  ^*∗*^ ^*∗*^ ^*∗*^Wilcoxon test. ^a^For the control group, *p* ≤ 0.05^a1^, *p* ≤ 0.01^a2^, *p* ≤ 0.001^a3^. ^c^For the HFCS + omega-3 fatty acids group, *p* ≤ 0.05^c1^, *p* ≤ 0.01^c2^, *p* ≤ 0.001^c3^. ^b^For the HFCS group, *p* ≤ 0.05^b1^, *p* ≤ 0.01^b2^, *p* ≤ 0.001^b3^. ^d^For the HFCS + probiotic group, *p* ≤ 0.05^d1^, *p* ≤ 0.01^d2^, *p* ≤ 0.001^d3^. Bold shows statistically significant values.

**Table 3:** Weekly fluid intake levels in rats.

Fluid intake levels (mL)						
Weeks	Control group (*n* = 8)	HFCS group (*n* = 8)	HFCS + omega-3 fatty acids group (*n* = 8)	HFCS + probiotic group (*n* = 8)	HFCS + omega-3 fatty acids + probiotic group (*n* = 8)	*p* ^*∗*^
	Mean ± SD	Mean ± SD	Mean ± SD	Mean ± SD	Mean ± SD	
1	282.57 ± 10.29	304.42 ± 19.07^a2^ ^*∗*^ ^*∗*^	294.28 ± 12.91	287.71 ± 13.02	294.14 ± 10.62	0.080
2	292.28 ± 15.39	317.85 ± 18.10^a1^ ^*∗*^ ^*∗*^	285.71 ± 14.97^b3^ ^*∗*^ ^*∗*^	296.28 ± 10.54^b2^ ^*∗*^ ^*∗*^	289.85 ± 16.96^b2^ ^*∗*^ ^*∗*^	**0.006**
*p* ^*∗∗∗*^	0.173	**0.017**	0.446	0.063	0.612	
3	294.28 ± 14.84	331.57 ± 15.76^a3^ ^*∗*^ ^*∗*^	293.42 ± 15.39^b3^ ^*∗*^ ^*∗*^	292.00 ± 15.08^b3^ ^*∗*^ ^*∗*^	290.14 ± 15.18^b3^ ^*∗*^ ^*∗*^	**0.003**
*p* ^*∗∗∗*^	0.672	**0.027**	0.228	0.518	0.752	
4	297.85 ± 13.56	340.28 ± 15.76^a3^ ^*∗*^ ^*∗*^	281.57 ± 10.99^a1.b3^ ^*∗*^ ^*∗*^	278.71 ± 10.35^a2.b3^ ^*∗*^ ^*∗*^	279.71 ± 11.70^a1.b3^ ^*∗*^ ^*∗*^	**0.001**
*p* ^*∗∗∗*^	0.271	**0.028**	0.128	0.064	0.204	
5	293.14 ± 10.05	321.42 ± 18.67^a1^ ^*∗*^ ^*∗*^	276.42 ± 10.17^a2.b3^ ^*∗*^ ^*∗*^	272.85 ± 13.09^a3.b3^ ^*∗*^ ^*∗*^	275.00 ± 13.16^a2.b3^ ^*∗*^ ^*∗*^	**0.001**
*p* ^*∗∗∗*^	0.310	0.176	0.176	0.237	0.310	
6	298.14 ± 11.69	337.28 ± 18.63^a1^ ^*∗*^ ^*∗*^	278.00 ± 10.27^a3.b3^ ^*∗*^ ^*∗*^	277.85 ± 15.75^a1.b3^ ^*∗*^ ^*∗*^	281.28 ± 13.02^a1.b3^ ^*∗*^ ^*∗*^	**0.001**
*p* ^*∗∗∗*^	0.236	**0.018**	0.799	0.463	0.293	
7	301.57 ± 12.50	341.85 ± 15.91^a1^ ^*∗*^ ^*∗*^	277.42 ± 9.27^a3.b3^ ^*∗*^ ^*∗*^	270.14 ± 12.03^a3.b3^ ^*∗*^ ^*∗*^	272.71 ± 9.92^a3.b3^ ^*∗*^ ^*∗*^	**0.001**
*p* ^*∗∗∗*^	0.735	0.345	0.674	0.249	0.108	

^*∗*^Kruskal–Wallis H-test.  ^*∗*^ ^*∗*^Mann–Whitney *U* test.  ^*∗*^ ^*∗*^ ^*∗*^Wilcoxon test. ^a^For the control group, *p* ≤ 0.05^a1^, *p* ≤ 0.01^a2^, *p* ≤ 0.001^a3^. ^b^For the HFCS group, *p* ≤ 0.05^b1^, *p* ≤ 0.01^b2^, *p* ≤ 0.001^b3^. Bold shows statistically significant values.

**Table 4:** Changes in body and liver weights of rats.

Weight (g)	Control group (*n* = 8)	HFCS group (*n* = 8)	HFCS + omega-3 fatty acids group (*n* = 8)	HFCS + probiotic group (*n* = 8)	HFCS + omega-3 fatty acids + probiotic group (*n* = 8)	*p∗*
	Mean ± SD	Mean ± SD	Mean ± SD	Mean ± SD	Mean ± SD	
Initial weight	265.75 ± 47.01	263.75 ± 38.96	261.25 ± 28.57	251.50 ± 28.73	275.75 ± 32.65	0.756
Third-week weight	288.12 ± 47.64	296.25 ± 38.10	303.62 ± 28.96	285.25 ± 30.16	315.87 ± 32.79	0.453
*p* ^*∗∗∗*^	**0.004**	**0.006**	**0.007**	**0.002**	**0.012**	
Sacrifice weight	324.25 ± 49.96	346.00 ± 37.06	313.75 ± 38.78	303.55 ± 21.42	299.50 ± 48.12	0.170
*p* ^*∗∗∗*^	**0.015**	**0.019**	0.123	**0.017**	**0.007**	
1^st^ weight change (%)	8.67 ± 1.31	12.62 ± 2.11^a2^*∗∗*	16.38 ± 1.81^a3,b3^*∗∗*	13.53 ± 1.30^a3^*∗∗*	14.76 ± 2.11^a3,b1^*∗∗*	**0.001**
2^nd^ weight change (%)	12.73 ± 3.84	17.15 ± 5.68	3.16 ± 6.23^a3,b3^*∗∗*	6.76 ± 4.46^a1,a3,b3^*∗∗*	−5,67 ± 6.78^a3,b3,d3^*∗∗*	**0.001**
*p∗∗∗*	**0.018**	**0.034**	**0.001**	**0.001**	**0.001**	
3^rd^ weight change (%)	22.45 ± 4.66	31.96 ± 7.97^a2^*∗∗*	20.04 ± 7.04^b3^*∗∗*	21.25 ± 6.20^b2^*∗∗*	8.15 ± 6.42^a3,b3,c3,d3^*∗∗*	**0.001**
*p∗∗∗*	**0.001**	**0.001**	**0.001**	**0.001**	**0.001**	
Liver weight	10.75 ± 1.03	11.87 ± 1.35	10.12 ± 1.7^b1^*∗∗*	9.62 ± 1.76^b1^*∗∗*	9.87 ± 1.95^b3,c3^*∗∗*	**0.049**
Liver index	3.34 ± 0.26	3.43 ± 0.09	3.21 ± 0.23	3.15 ± 0.38	3.28 ± 0.24	0.236

1^st^ weight change (%): (third-week weight − initial weight)/initial weight) *×* 0100. 2^nd^ weight change (%): (sacrifice weight − third-week weight)/third-week weight) *×* 100. 3^rd^ weight change (%): (sacrifice weight − initial weight)/initial weight) *×* 100. Liver index: (liver weight/sacrifice weight) *×* 100. One-way ANOVA.*∗∗*Unpaired *t*-test. *∗∗∗*Paired *t*-test. ^a^For the control group, *p* ≤ 0.05^a1^, *p* ≤ 0.01^a2^, *p* ≤ 0.001^a3^. ^b^For the HFCS group, *p* ≤ 0.05^b1^, *p* ≤ 0.01^b2^, *p* ≤ 0.001^b3^. ^c^For the HFCS + omega-3 fatty acids, *p* ≤ 0.05^c1^, *p* ≤ 0.01^c2^, *p* ≤ 0.001^c3^. ^d^For the HFCS + probiotic group, *p* ≤ 0.05^d1^, *p* ≤ 0.01^d2^, *p* ≤ 0.001^d3^. Bold shows statistically significant values.

**Table 5:** Glucose, liver enzymes, serum , and triglyceride levels of rats.

Parameters	Control group (*n* = 8)	HFCS group (*n* = 8)	HFCS + omega-3 fatty acids group (*n* = 8)	HFCS + probiotic group (*n* = 8)	HFCS + omega-3 fatty acids + probiotic group (*n* = 8)	*p∗*
	Mean ± SD	Mean ± SD	Mean ± SD	Mean ± SD	Mean ± SD	
Glucose (mg/dL)	132.98 ± 21.09	164.73 ± 15.78^a2^*∗∗*	136.73 ± 15.90^b2^*∗∗*	141.92 ± 21.52^b1^*∗∗*	142.67 ± 24.21^b1^*∗∗*	**0.031**
	(94.50–155.00)	(149.70–197.40)	(117.50–162.00)	(109.30–175.00)	(110.00–178.00)	
ALT (U/L)	38.32 ± 7.21	67.06 ± 10.42^a3^*∗∗*	33.28 ± 5.09^b3^*∗∗*	28.67 ± 6.54^a1.b3^*∗∗*	37.23 ± 8.52^b3.d1^*∗∗*	**0.001**
	(29.00–50.80)	(54.80–85.70)	(24.70–41.50)	(21.90–39.80)	(27.20–50.00)	
AST (U/L)	123.10 ± 68.49	169.38 ± 32.30^a1^*∗∗*	118.37 ± 17.34^b3^*∗∗*	97.63 ± 26.89^b3^*∗∗*	121.15 ± 16.36^b3^*∗∗*	**0.003**
	(81.50–282.50)	(128.50–226.70)	(97.30–152.40)	(68.60–146.90)	(90.60–141.50)	
ALP (U/L)	96.75 ± 22.95	99.12 ± 23.28	99.00 ± 18.67	90.25 ± 16.85	98.50 ± 13.85	0.645
	(70.00–138.00)	(54.00–131.00)	(80.00–126.00)	(62.00–119.00)	(71.00–113.00)	
Total cholesterol (mg/dL)	27.13 ± 5.39	40.22 ± 6.43^a3^*∗∗*	32.55 ± 3.94^b2^*∗∗*	33.86 ± 4.09^a1.b1^*∗∗*	29.55 ± 2.49^b3^*∗∗*	**0.001**
	(19.70–33.90)	(33.60–51.30)	(28.70–40.20)	(29.00–38.90)	(26.10–32.40)	
Triglyceride (mg/dL)	36.01 ± 13.37	60.60 ± 16.73^a2^*∗∗*	40.15 ± 10.84^b2^*∗∗*	44.46 ± 9.87^b1^*∗∗*	42.35 ± 25.87^b1^*∗∗*	**0.049**
	(18.00–51.70)	(32.00–82.00)	(23.70–62.30)	(26.70–60.60)	(20.70–100.90)	

*∗*Kruskal–Wallis H-test. *∗∗*Mann–Whitney *U* test. ^a^For the control group, *p* ≤ 0.05^a1^, *p* ≤ 0.01^a2^, *p* ≤ 0.001^a3^. ^b^For the HFCS group, *p* ≤ 0.05^b1^, *p* ≤ 0.01^b2^, *p* ≤ 0.001^b3^. ^d^For the HFCS + probiotic group, *p* ≤ 0.05^d1^, *p* ≤ 0.01^d2^, *p* ≤ 0.001^d3^. Bold shows statistically significant values.

**Table 6:** Liver tissue triglycerides, total cholesterol, and total protein contents of rats.

Parameters	Control group (*n* = 8)	HFCS group (*n* = 8)	HFCS + omega-3 fatty acids group (*n* = 8)	HFCS + probiotic group (*n* = 8)	HFCS + omega-3 fatty acids + probiotic group (*n* = 8)	*p∗*
	Mean ± SD	Mean ± SD	Mean ± SD	Mean ± SD	Mean ± SD	
Triglyceride (*μ*mol/g tissue)	6.08 ± 0.91	11.16 ± 1.55^a3^*∗∗*	8.26 ± 0.91^a3,b3^*∗∗*	8.21 ± 1.38^a3,b2^*∗∗*	7.88 ± 1.23^a2,b3^*∗∗*	**0.001**
	(4.94–7.66)	(9.32–13.61)	(6.94–9.57)	(6.68–10.75)	(5.45–9.81)	
Total cholesterol (*μ*mol/g tissue)	1.78 ± 0.63	3.23 ± 0.39^a3^*∗∗*	1.94 ± 0.37^b3^*∗∗*	1.86 ± 0.32^b3^*∗∗*	1.82 ± 0.23^b3^*∗∗*	**0.001**
	(0.90–2.92)	(2.69–3.79)	(1.45–2.46)	(1.47–2.33)	(1.51–2.12)	
Total protein (mg/g protein)	5.81 ± 1.48	8.02 ± 1.05^a3^*∗∗*	6.01 ± 0.65^b3^*∗∗*	5.61 ± 0.80^b3^*∗∗*	5.76 ± 0.31^b3^*∗∗*	**0.002**
	(3.24–7.38)	(6.49–9.31)	(4.86–6.91)	(4.56–6.45)	(5.17–6.10)	

*∗*One-way ANOVA. *∗∗*Unpaired *t*-test. ^a^For the control group, *p* ≤ 0.05^a1^, *p* ≤ 0.01^a2^, *p* ≤ 0.001^a3^. ^b^For the HFCS group, *p* ≤ 0.05^b1^, *p* ≤ 0.01^b2^, *p* ≤ 0.001^b3^. Bold shows statistically significant values.

**Table 7:** Scoring the level of steatosis in liver tissue of rats.

Microscopic scoring	Control group (*n* = 8)	HFCS group (*n* = 8)	HFCS + omega-3 fatty acids group (*n* = 8)	HFCS + probiotic group (*n* = 8)	HFCS + omega-3 fatty acids + probiotic group (*n* = 8)	*p∗*
	Mean ± SD	Mean ± SD	Mean ± SD	Mean ± SD	Mean ± SD	
Steatosis (0–3)	0.18 ± 0.11	2.05 ± 0.17^a3^*∗∗*	0.95 ± 0.12^a3,b3^*∗∗*	0.81 ± 0.10^a3,b3,c2^*∗∗*	0.72 ± 0.11^a3,b3,c2,d1^*∗∗*	**0.001**
	(0.10–0.42)	(1.80–2.33)	(0.84–1.24)	(0.65–1.02)	(0.62–0.98)	
Lobular inflammation (0–3)	0.00 ± 0.00	1.08 ± 0.12^a3^*∗∗*	0.21 ± 0.10^a3,b3^*∗∗*	0.13 ± 0.05^a3,b3^*∗∗*	0.11 ± 0.02^a3,b3,c1^*∗∗*	**0.001**
	(0.00–0.00)	(0.92–1.31)	(0.11–0.43)	(0.10–0.27)	(0.10–0.17)	
Hepatocellular ballooning (0–2)	0.00 ± 0.00	0.27 ± 0.06^a3^*∗∗*	0.00 ± 0.00^b3^	0.00 ± 0.00^b3^	0.00 ± 0.00^b3^	**0.001**
	(0.00–0.00)	(0.21–0.43)	(0.00–0.10)	(0.00–0.00)	(0.00–0.00)	
NAS(0–8)	0.18 ± 0.11	3.41 ± 0.24^a3^*∗∗*	1.23 ± 0.13^a3,b3^*∗∗*	0.94 ± 0.12^a3,b3,c3^*∗∗*	0.84 ± 0.13^a3,b3,c3^*∗∗*	**0.001**
	(0.10–0.42)	(3.10–3.73)	(1.11–1.45)	(0.76–1.15)	(0.73–1.11)	

*∗*Kruskal–Wallis H-test. *∗∗*Mann–Whitney *U* test. ^a^For the control group, *p* ≤ 0.05^a1^, *p* ≤ 0.01^a2^, *p* ≤ 0.001^a3^. ^b^For the HFCS group, *p* ≤ 0.05^b1^, *p* ≤ 0.01^b2^, *p* ≤ 0.001^b3^. ^c^For the HFCS + omega-3 fatty acids group, *p* ≤ 0.05^c1^, *p* ≤ 0.01^c2^, *p* ≤ 0.001^c3^. ^d^For the HFCS + probiotic, *p* ≤ 0.05^d1^, *p* ≤ 0.01^d2^, *p* ≤ 0.001^d3^. Bold shows statistically significant values.

## Data Availability

The data used to support the findings of this study are available from the corresponding author upon request.

## Additional Points

Supplements were demonstrated in the fatty liver model induced by HFCS. From a public health perspective, due to the rapid growth of fatty liver disease worldwide and the increasing use of high-fructose corn syrup, especially in the composition of foods, this disease becomes alarming every passing day. Today, the fundamental grounds of treatment are based on nutrition therapy, weight loss, and exercise. However, these interventions fail to be permanent, and their effectiveness is low. Probiotic and omega-3 fatty acids reduced hepatic steatosis content especially when combined with nutritional intervention. Probiotic and omega-3 fatty acid supplements may have a protective effect in the fatty liver model induced by HFCS. A high-fructose diet and an omega-3 fatty acids-deficient diet are thought to play an important role in the development of NAFLD. Supplements have a beneficial effect on some liver tissue parameters important in fatty liver and weight management.
